# Comparative Efficacy and Acceptability of Transdiagnostic Psychotherapies for Emotional Disorders: Protocol for a Network Meta-Analysis of Randomized Controlled Trials

**DOI:** 10.2196/104791

**Published:** 2026-10-06

**Authors:** Qiaosi Cai, Yihan Zeng, Noelia Jiménez-Orenga, Clara Miguel, Mathias Harrer, Pim Cuijpers, Amanda Díaz-García, Qun Ye

**Affiliations:** 1 Zhejiang Key Laboratory of Intelligent Education Technology and Application, Zhejiang Philosophy and Social Science Laboratory for the Mental Health and Crisis Intervention of Children and Adolescents School of Psychology Zhejiang Normal University Jinhua, Zhejiang China; 2 Universitat Jaume I Castellón de la Plana, Castellón Spain; 3 Vrije Universiteit Amsterdam Amsterdam The Netherlands; 4 Universidad de Zaragoza Teruel Spain

**Keywords:** transdiagnostic treatment, cognitive behavioral therapy, emotional disorders, anxiety, depression, psychotherapy, network meta-analysis

## Abstract

**Background:**

Emotional disorders—primarily anxiety and unipolar depressive disorders—are highly prevalent, disabling, and frequently comorbid. Disorder‑specific cognitive behavioral therapy (DS‑CBT) is considered a gold standard treatment, yet it can be inefficient in the context of high comorbidity and service constraints. Transdiagnostic psychotherapies that target shared maintaining mechanisms (eg, emotion dysregulation and neuroticism) have been developed, including the Unified Protocol, transdiagnostic behavior therapy, emotion regulation therapy, and various transdiagnostic internet-delivered cognitive behavioral therapy protocols. Recent meta-analyses suggest that transdiagnostic interventions are effective or likely effective, but a comprehensive comparative evaluation across different transdiagnostic protocols, DS‑CBT, other evidence‑based psychotherapies, and control conditions is lacking.

**Objective:**

This study aims to conduct a network meta-analysis (NMA) to compare the efficacy and acceptability of various transdiagnostic therapies (eg, the Unified Protocol, transdiagnostic behavior therapy, and emotion regulation therapy), DS-CBT, other evidence-based active therapies (eg, acceptance and commitment therapy, behavioral activation, and mindfulness-based cognitive therapy), and control conditions (waitlist or care as usual) in improving symptoms in adults with anxiety and/or depressive disorders and generate a probabilistic ranking of their efficacy.

**Methods:**

This meta-analysis will include randomized controlled trials from PubMed, Embase, PsycInfo, and the Cochrane Library, complemented by existing meta‑analytic research domain databases for depression and anxiety. Eligible participants are adults (≥18 years) with a principal diagnosis of anxiety and/or unipolar depressive disorder according to established diagnostic classification systems (eg, any version of the *Diagnostic and Statistical Manual of Mental Disorders* or *International Classification of Diseases*, 10th or 11th revisions) or scoring above a validated cutoff on anxiety or depression scales. Primary outcomes are continuous measures of anxiety and depressive symptom severity at the posttreatment time point; secondary outcomes include study dropout (acceptability) and response or remission where available. We will perform random-effects network meta-analyses within a frequentist framework using the *netmeta* package in R to estimate relative treatment effects and *P* scores for treatment rankings and examine transitivity and consistency. We will conduct additional Bayesian NMAs as sensitivity analyses using the surface under the cumulative ranking curve for ranking.

**Results:**

As of September 2026, the literature search has been completed, and title and abstract screening is ongoing. Data analysis will be conducted in late 2026. We expect the meta-analysis to be completed and submitted for publication by the end of 2026, with results expected to be published in early 2027.

**Conclusions:**

This NMA will provide comprehensive comparative evidence on the efficacy and acceptability of transdiagnostic psychotherapies for emotional disorders, which can inform clinical decision-making and guideline development.

**Trial Registration:**

OSF Registries osf.io/p8957; https://osf.io/p8957/overview

**International Registered Report Identifier (IRRID):**

DERR1-10.2196/104791

## Introduction

### Background

Emotional disorders (EDs) represent a spectrum of conditions characterized by frequent, intense negative emotional experiences and maladaptive responses to such experiences [1]. The term commonly encompasses anxiety disorders (generalized anxiety disorder [GAD], social anxiety disorder [SAD], panic disorder, agoraphobia, and specific phobias) and unipolar depressive disorders (major depressive disorder and persistent depressive disorder) [2]. Data from the Global Burden of Disease study show that depressive and anxiety disorders are the 2 most disabling mental disorders worldwide [3,4].

A critical challenge in treating EDs is the exceptionally high comorbidity between anxiety and depression [5]. A large-scale survey revealed that among individuals with a lifetime history of major depressive episodes, 51% also had a lifetime diagnosis of any anxiety disorder, whereas among those without major depressive episodes, the lifetime prevalence of any anxiety disorder was only 11.8% [6]. This high rate of comorbidity underscores that diagnosis-specific treatment models are increasingly inadequate for addressing the current mental health crisis [7], highlighting an urgent clinical need for transdiagnostic intervention approaches based on shared mechanisms [8].

The current gold standard treatment—disorder-specific cognitive behavioral therapy (DS-CBT)—was designed to address single disorders [9]. When confronted with comorbidity, clinicians face the dilemma of determining which disorder to prioritize or are compelled to deliver sequential protocols, resulting in prolonged treatment duration, increased costs, and reduced efficiency [8].

In response to these limitations, transdiagnostic psychotherapies have emerged as an innovative approach. Grounded in the theoretical premise that comorbidities arise from shared underlying mechanisms—such as neuroticism, experiential avoidance, and emotion regulation deficits [10,11]—transdiagnostic psychotherapies are designed to simultaneously address symptoms across multiple EDs. The Unified Protocol (UP) for transdiagnostic treatment of EDs [11], transdiagnostic behavior therapy (TBT) [12], emotion regulation therapy (ERT) [13], and transdiagnostic internet-delivered cognitive behavioral therapy (iCBT) [14] exemplify this paradigm shift.

Recent meta‑analyses have synthesized parts of this rapidly growing literature: Jiménez‑Orenga et al [2] pooled 94 randomized controlled trials (RCTs; 12,443 participants) testing broader transdiagnostic psychological interventions for EDs, finding moderate effects (*g*≈0.6) vs control that were robust across sensitivity analyses. Cuijpers et al [15] meta‑analyzed 45 RCTs of transdiagnostic interventions for depression and/or anxiety, reporting moderate effects (*g*≈0.5) at the posttreatment time point, with effects attenuating at longer follow‑up. Schaeuffele et al [16] focused specifically on unified transdiagnostic CBT (TD‑CBT) treatments and found that TD‑CBT showed large pretest‑to‑posttest effects and was comparable to DS‑CBT, with sustained benefits up to 24 months for internet-based TD‑CBT.

However, these meta‑analyses did not fully address comparative efficacy across specific transdiagnostic protocols (eg, UP vs TBT vs ERT) or systematically situate TD‑CBT relative to DS‑CBT and other bona fide psychotherapies using both direct and indirect evidence. Furthermore, prior work has typically been restricted either to CBT-based transdiagnostic interventions [16], a single delivery format (eg, iCBT) [17], or transdiagnostic interventions broadly defined irrespective of theoretical orientation [2].

### Aims and Objectives

A network meta-analysis (NMA) can combine direct and indirect comparisons to estimate relative treatment effects and rank multiple interventions simultaneously within either a frequentist or a Bayesian framework. This study will conduct primary analyses within a frequentist framework using the *netmeta* [18] package in R (R Foundation for Statistical Computing). Building on existing meta-analytic research domains for depression and anxiety psychotherapies [19] and on the most recent TD‑CBT and transdiagnostic psychological intervention meta‑analyses, this study aims to provide a comprehensive comparative evaluation of transdiagnostic psychotherapies for EDs. The primary objective of this NMA is to compare the short‑term efficacy (after treatment) of transdiagnostic protocols (UP, TBT, ERT, and other unified TD‑CBTs), DS‑CBT, other evidence‑based psychotherapies (EBPs; eg, behavioral activation [BA] and problem-solving therapy [PST]), and control conditions (waitlist or care as usual [CAU]) on continuous measures of anxiety and depressive symptom severity in adults with EDs.

The secondary objectives are to (1) compare the acceptability of these interventions as indexed by all‑cause study dropout (ie, discontinuation from the study for any reason); (2) explore potential effect modifiers (eg, delivery format and baseline severity) using subgroup analyses and, where feasible, network meta-regression; and (3) grade the certainty of evidence for key comparisons using a Grading of Recommendations Assessment, Development, and Evaluation (GRADE)–compatible framework.

## Methods

This protocol follows the PRISMA-NMA (Preferred Reporting Items for Systematic Reviews and Meta-Analyses extension for network meta-analyses) guidance and has been registered on the Open Science Framework [20].

### Searches in Bibliographical Databases

Following the methodological framework established in previous high-impact NMAs in this field [2,15], we conducted independent searches in PubMed, Embase, PsycInfo, and the Cochrane Library. The search strategy combined terms related to 3 key concepts using Boolean operators: population (eg, “emotional disorders,” “anxiety,” “depression,” “generalized anxiety disorder,” “social anxiety disorder,” “panic disorder,” and “major depressive disorder”), intervention (eg, “transdiagnostic,” “unified protocol,” “emotion regulation therapy,” “cognitive behavioral therapy,” “CBT,” “acceptance and commitment therapy,” and “behavioral activation”), and study design (eg, “randomized controlled trial” and “RCT”). The full search strategies for each database are provided in Multimedia Appendix 1. The search was adapted for each database according to its specific syntax and indexing. In addition, we supplemented the primary searches using the meta-analytic research domain databases within the Metapsy project [21] to cross-validate our results and reduce the risk of missing relevant trials. To further supplement our search, we will manually screen the reference lists of all included studies and relevant systematic reviews. Only studies published in English were included because of resource constraints. We will acknowledge the potential impact of this restriction as a limitation and document the studies excluded for this reason.

### Inclusion and Exclusion of Studies

Eligibility criteria were defined using the population, intervention, comparison, outcome, and study design framework.

#### Participants

Participants must be adults aged 18 years or older recruited from any source, including community or outpatient clinic populations. Inclusion is determined according to the following criteria: (1) studies with participants explicitly identified as a mixed anxiety population or a mixed anxiety-depression population will be directly eligible for inclusion; (2) studies with participants diagnosed with depression alone without any mention of anxiety conditions will be generally excluded unless the participants’ scores on an initial anxiety scale exceed the clinical cutoff for that scale, in which case the studies will be included; (3) studies with participants diagnosed with a specific anxiety disorder alone (eg, SAD or GAD) without any mention of depression or other anxiety disorders will be generally excluded unless the participants’ scores on an initial depression scale or another anxiety scale exceed the corresponding clinical cutoff, in which case the studies will be included; (4) studies with participants screened via self-report measures whose scores exceed the clinical cutoff on at least 2 relevant emotional dimensions (eg, 2 distinct anxiety disorders, one anxiety disorder combined with depression, or a general emotional distress scale) will be included; and (5) trials explicitly stating that the intervention is a transdiagnostic therapy—defined as a therapy targeting multiple EDs—will be included regardless of participants’ specific diagnoses. The following exclusion criteria also apply: (1) trials in which more than 10% of the total sample comprises patients with disorders previously classified as anxiety disorders, specifically obsessive-compulsive disorder and posttraumatic stress disorder, will be excluded (note that, following the *Diagnostic and Statistical Manual of Mental Disorders, Fifth Edition*, reclassification, obsessive-compulsive disorder and posttraumatic stress disorder are no longer categorized as anxiety disorders); and (2) trials that explicitly indicate that they target participants with somatic or physical diseases will be excluded.

#### Interventions and Control Conditions

In this NMA, nodes will be defined based on each distinct psychotherapeutic intervention (eg, UP, TBT, ERT, acceptance and commitment therapy [ACT], and mindfulness-based cognitive therapy [MBCT]) as reported in the included studies. Node assignment will be based primarily on how the original trial authors describe and label the intervention rather than on our own theoretical judgment of the therapy’s scope. If the number of studies for a particular intervention is insufficient to support stable estimation, we will consider aggregating nodes based on shared theoretical foundations (eg, grouping similar TD-CBT interventions or aggregating third‑wave therapies) as a post hoc sensitivity analysis, and any such aggregation will be clearly justified and reported.

Transdiagnostic psychotherapies are manualized, standardized psychological interventions that treat related disorders by targeting shared transdiagnostic mechanisms underlying them. Notable examples include the UP, a transdiagnostic cognitive behavioral intervention that addresses the core underlying mechanism common to EDs (namely, neuroticism and maladaptive emotion regulation strategies); TBT; ERT; false safety behavior elimination therapy; affect regulation training; and other unified broadband protocols.

DS-CBT comprises standard protocols for a principal diagnosis of GAD, SAD, panic disorder, agoraphobia, specific phobias, or major depressive disorder.

Other EBPs are active, structured psychotherapies with established evidence, including third-wave therapies (eg, ACT and MBCT), behavioral therapies (eg, BA and PST), psychodynamic therapy, and interpersonal therapy.

Control conditions are nonactive or minimal intervention conditions, including waitlist and CAU.

#### Comparators

This NMA will include studies comparing any 2 treatment conditions defined in the previous section. Accordingly, the following types of comparisons are eligible: (1) comparisons involving a control condition (studies comparing any active psychotherapy [eg, transdiagnostic psychotherapies, DS-CBT, or other EBPs] to a control condition) and (2) comparisons between 2 active psychotherapies (studies comparing one active psychotherapy to another active psychotherapy, which may include but is not limited to transdiagnostic psychotherapies vs DS-CBT, transdiagnostic psychotherapies vs other EBPs, DS-CBT vs other EBPs, and head-to-head comparisons among different transdiagnostic psychotherapies).

#### Outcomes

The primary outcomes (continuous measures assessed at the posttreatment time point) are (1) depression severity, measured via validated self-report or clinician-rated scales (eg, Beck Depression Inventory or Beck Depression Inventory–II [BDI-II], Patient Health Questionnaire–9 [PHQ-9], Montgomery-Åsberg Depression Rating Scale, Hamilton Depression Rating Scale, Hospital Anxiety and Depression Scale [HADS] depression subscale, and Overall Depression Severity and Impairment Scale); (2) anxiety severity, measured via validated self-report or clinician-rated scales (eg, Beck Anxiety Inventory [BAI], Generalized Anxiety Disorder–7 [GAD-7], HADS anxiety subscale, Overall Anxiety Severity and Impairment Scale, State-Trait Anxiety Inventory–Trait subscale, and Hamilton Anxiety Rating Scale); and (3) composite emotional symptom severity (where available), measured using scales that jointly assess anxiety and depression (eg, HADS total score and Patient Health Questionnaire Anxiety and Depression Scale) to align with previous transdiagnostic psychotherapy meta-analyses.

Secondary outcomes are (1) all‑cause study dropout up to the posttreatment time point (acceptability), extracted as the number of participants randomized who did not provide posttreatment data for any reason; (2) response and remission rates where consistently defined (eg, ≥50% symptom reduction; score below the threshold); and (3) remission of a secondary or nontargeted eligible anxiety or depressive disorder where reported and defined. Data will be synthesized separately if definitions and measures are sufficiently comparable across studies; otherwise, they will be summarized narratively.

The time points are at posttreatment (assessments closest to treatment end within –2 weeks to +2 weeks).

#### Study Design

Only RCTs will be included.

### Study Selection

We will import all retrieved studies into EndNote (Clarivate Analytics) for initial deduplication, followed by secondary deduplication using Rayyan (Rayyan Systems Inc). For title and abstract screening, we will first randomly select a calibration set comprising 10% of the total identified references after deduplication to train and calibrate large language models (LLMs). The LLMs we will use for screening are DeepSeek‑V4‑Pro‑Think and Claude Sonnet 4.6 Thinking (Anthropic). Meanwhile, 2 reviewers will independently screen the calibration set, and their decisions will serve as the reference standard. We will evaluate each LLM’s performance against human decisions using accuracy, sensitivity, and specificity. The LLM screening pipeline will be considered satisfactory if (1) each LLM achieves 90% agreement or more with human reviewers on the calibration set and (2) the agreement between the 2 LLMs (ie, proportion of records for which both models reach the same inclusion or exclusion decision) is of 95% or higher. Only after reaching this threshold will we deploy the LLMs to assist with the remaining title and abstract screening. During deployment, if both LLMs agree on a decision (include or exclude), that decision will be provisionally recorded. If the 2 LLMs disagree, the record will be flagged for human review. Human reviewers will verify all LLM-assisted outputs (ie, every record flagged by either LLM plus all disagreement records) and resolve any discrepancies. No final eligibility decision will be based solely on the LLMs. During the full‑text screening stage, 2 independent human reviewers will retrieve and assess the full texts of all potentially eligible studies against the inclusion and exclusion criteria. Disagreements will be resolved through discussion or consultation with a third reviewer. This LLM-assisted screening pipeline builds on similar methodological frameworks recently developed and validated in systematic reviews within the psychological literature [22]. We will use a PRISMA (Preferred Reporting Items for Systematic Reviews and Meta-Analyses) flow diagram to document the selection process.

### Data Extraction

Two independent reviewers will extract data from each included study using a standardized data extraction form. Disagreements will be resolved through discussion or consultation with a third reviewer.

#### Study-Level Characteristics

These will comprise first author, publication year, country, journal, and study design (eg, individual vs cluster randomization and number of arms).

#### Participant Characteristics

These will comprise the inclusion criteria (diagnosis vs symptom cutoff and which EDs were included); mean age, age range, and sex distribution; and recruitment source (community, clinical referrals, or other).

#### Intervention Characteristics

These will comprise treatment type and protocol name (UP, TBT, ERT, DS‑CBT, ACT, MBCT, BA, PST, or other), study protocol publication status, treatment manual availability, optional modules, session sequence and flexibility, session duration, total treatment duration, treatment format (individual vs group vs mixed), delivery mode (face-to-face, online guided, blended, videoconference, etc), treatment provider, and dropout rates.

#### Comparator Characteristics

These will comprise type of control (waitlist or CAU) and description of usual care where applicable.

#### Outcome Data

These will comprise (1) outcome instrument used (eg, BDI-II, PHQ-9, BAI, or GAD-7), the corresponding means and SDs (or change scores and response and remission rates) for primary and secondary outcomes at the posttreatment time point by arm, and effect size data if directly reported (Cohen *d* and Hedges *g*); (2) the number of randomized participants and number analyzed per arm (total, mean, and SD or SE and 95% CI); and (3) the number of study dropouts by arm and reasons if reported.

### Risk-of-Bias Assessment

Two independent reviewers will assess the risk of bias for each included study using version 2 of the Cochrane risk of bias tool for randomized trials (RoB 2) [23] following the operationalization and guidance developed by the Metapsy initiative for psychotherapy trials [24]. Disagreements will be resolved through discussion or consultation with a third reviewer.

### Effect Size Calculation

For continuous outcomes, we will compute the Hedges *g* (small-sample bias-corrected standardized mean differences [SMDs]) between groups at the posttreatment time point using posttreatment means and SDs where available and, if necessary, change scores, SEs, CIs, or test statistics. Where multiple anxiety or depression scales are reported, we will prioritize the scale designated as primary by the authors; if not specified, we will prioritize widely used, psychometrically robust measures (eg, BDI-II, PHQ-9, BAI, and GAD-7) and will conduct sensitivity analyses including alternative measures. All Hedges *g* values will be consistently coded so that negative values indicate greater symptom reduction for the more active treatment relative to the comparator. For binary outcomes (eg, response, remission, and dropout), we will calculate log relative risks and their sampling variances. When a study includes multiple relevant intervention arms, we will avoid double counting by (1) using appropriate multilevel hierarchical (pairwise) meta-analysis models or (2) using multi-arm NMA models that account for within-study correlations. For studies reporting more than one active transdiagnostic arm, each arm will be assigned to the most appropriate treatment node based on its intervention characteristics and protocol description.

### NMA

#### Network Geometry

We will first describe and visualize the geometry of the treatment network using the *netmeta* [18] package in R. In the network plot, nodes represent interventions, and edges represent direct comparisons. Node size will be proportional to the number of participants, and edge thickness will be proportional to the number of trials. Nodes will be color coded by treatment class to enhance interpretability. Additionally, we will generate a network contribution plot to illustrate the contribution of direct evidence to each network estimate and a net heat plot to identify potential inconsistency hot spots where applicable.

#### NMA Model

##### Overview

We will conduct random-effects NMAs primarily within a frequentist framework using the *netmeta* [18] package in R. In addition, we will conduct Bayesian NMAs as sensitivity analyses. The transitivity assumption will be assessed prior to analysis by comparing the distribution of potential effect modifiers across treatment comparisons.

##### Frequentist NMA (Primary Analysis)

Random-effects NMAs will be performed using the *netmeta* [18] function, which is based on a graph-theoretical approach to NMA. Effect sizes will be expressed as SMDs (Hedges *g*) with 95% CIs for continuous outcomes. For dichotomous efficacy outcomes (response and remission), odds ratios will be converted to the SMD scale using the transformation method reported by Chinn [25] and then pooled together with continuous outcomes in the same NMA. For acceptability (dropout), log risk ratios will be used in a separate NMA model as acceptability represents a conceptually distinct outcome. Between-study heterogeneity (τ^2^) will be estimated using restricted maximum likelihood, and a common τ^2^ will be assumed across all treatment comparisons. Global and local inconsistency will be evaluated using methods implemented in *netmeta* [18], including the design-by-treatment interaction model and the node-splitting (separate indirect from direct design evidence) method [26].

##### Bayesian NMA (Sensitivity Analyses)

As sensitivity analyses, we will conduct Bayesian random-effects NMAs using the *gemtc* [27] package in R (interfacing with Just Another Gibbs Sampler [28]). Given the anticipated clinical heterogeneity, a random-effects model will be prioritized. We will assign vague prior distributions for treatment effects (N[0, 10^2^]) and the between-study SD (uniform[0,5]). Four Markov chains will be run simultaneously with 50,000 iterations using a 10,000-iteration burn-in period and a thinning interval of 10. Convergence will be assessed using the Brooks-Gelman-Rubin diagnostic (potential scale reduction factor), where values below 1.05 indicate satisfactory convergence. If no stable CRAN version of *gemtc* [27] is available at the time of the analysis, the same model will be directly implemented using Just Another Gibbs Sampler or BUGS [28].

#### Ranking Probabilities

In the frequentist framework, *P* scores will be calculated to establish a hierarchy of treatment efficacy. In Bayesian sensitivity analyses, the surface under the cumulative ranking curve will be used instead. Higher values indicate a greater likelihood that a therapy is among the most effective. The certainty of the ranking will be quantified using the precision of treatment hierarchies approach as implemented in the *poth* package.

#### Subgroup Analysis and Meta-Regression

To explore sources of heterogeneity, the following prespecified effect modifiers will be examined where data are sufficient (defined as at least 5 studies per subgroup with adequate network connectivity to support meaningful estimation for subgroup analyses and at least 10 studies with complete covariate data for meta-regression).

Subgroup analyses (categorical moderators) will include (1) delivery format (face-to-face vs remote [delivered via smartphone apps, computer programs, or other online platforms] vs mixed [combining face-to-face and remote elements]), (2) treatment format (individual vs group), (3) guidance type (guided vs unguided), and (4) inclusion criteria (diagnosis based [eg, meeting *Diagnostic and Statistical Manual of Mental Disorders* or *International Classification of Diseases* criteria for a principal diagnosis] vs symptom cutoff based [scoring above validated thresholds on self-report measures]).

For categorical moderators, each subgroup variable will be entered separately as a categorical covariate in a network meta-regression model using the *netmeta* [18] function.

We will conduct network meta-regression to examine the impact of the following continuous covariates provided that the minimum study requirements are met: (1) baseline symptom severity (mean baseline score on the primary outcome measure), (2) proportion of female participants (percentage of female participants in the study sample), (3) mean study-level risk-of-bias score (aggregated RoB 2 score across domains), and (4) baseline proportion of participants with co-occurring eligible anxiety or depressive disorders.

For continuous moderators, a separate univariable random-effects meta-regression model will be fitted for each covariate using the *netmeta* [18] function, which estimates the regression coefficient while accounting for between-study heterogeneity. All meta-regression analyses will be considered exploratory; results will be reported as regression coefficients with 95% CIs and corresponding *P* values. Given the exploratory nature of these analyses, no adjustment for multiple testing will be applied, and findings will be interpreted with caution accordingly.

#### Heterogeneity and Small-Study Effects

In NMAs, we will report the estimated between‑study SD (τ) and consider its magnitude relative to benchmarks from psychotherapy meta-analyses (eg, τ=0.1-0.2 as small, τ=0.3-0.5 as moderate, and τ>0.5 as large). We will explore the impact of heterogeneity via sensitivity analyses and, where feasible, meta-regression. Comparison‑adjusted funnel plots will be used (if there are ≥10 studies per treatment comparison) to explore small‑study effects or publication bias in the network.

#### Certainty of Evidence

Following the methodology outlined by Cuijpers et al [29] and the Confidence in Network Meta-Analysis (CINeMA) framework [30], we will evaluate the certainty of evidence for each network estimate using the CINeMA web application according to the following 6 criteria: within-study bias, reporting bias, indirectness, imprecision, heterogeneity, and incoherence. Each comparison will be rated as being of high, moderate, low, or very low certainty, and summary tables with the confidence ratings will be presented.

#### Sensitivity Analyses

Preplanned sensitivity analyses include, first, analyses restricted to studies with low risk of bias. Second, analyses excluding interventions with potential transitivity violations will be conducted; interventions that are highly specific to a particular subpopulation (eg, therapies designed exclusively for older adults) may violate the transitivity assumption. Third, where Bayesian NMAs are conducted, comparison of results from the primary frequentist NMA with those from Bayesian models will be conducted to assess the robustness of treatment effect estimates and rankings.

### Ethical Considerations

This NMA involves secondary analyses of published aggregate data and does not require formal ethics approval. The findings will be disseminated via a peer‑reviewed journal article and conference presentations and will be shared with guideline developers and relevant professional societies.

## Results

As of September 2026, the literature search has been completed, and title and abstract screening is ongoing. Data analysis will be conducted in late 2026. We expect the meta-analysis to be completed and submitted for publication by the end of 2026, with results expected to be published in early 2027. The study was funded in September 2022 by the National Natural Science Foundation of China (grant 32200912).

## Discussion

This NMA aims to provide a comparative synthesis of the efficacy and acceptability of transdiagnostic psychotherapies for EDs. Its core methodological approach is to move beyond traditional pairwise comparisons by integrating direct and indirect evidence within a unified analytic framework, thereby enabling a comprehensive comparison across multiple transdiagnostic interventions. Relative to existing pairwise meta‑analyses, this approach offers 3 specific added values.

First, while prior work has often grouped transdiagnostic interventions broadly, we will directly compare specific protocols—including the UP, TBT, ERT, and various transdiagnostic iCBT protocols—using both direct and indirect evidence, allowing for head‑to‑head contrasts among these protocols as well as against DS‑CBT. This enables us to evaluate the relative merits of particular approaches, which is not feasible in pairwise meta‑analyses that collapse all transdiagnostic interventions together. Second, by including other EBPs alongside DS‑CBT and transdiagnostic psychotherapies in a single network, we can assess the positioning of TD‑CBT relative to DS‑CBT and other active therapies, providing a critical comparative basis for guideline development. Third, the NMA generates probabilistic treatment rankings and, combined with CINeMA‑graded certainty of evidence, offers an objective hierarchical evidence base for each intervention.

The findings may have important implications for clinical decision-making. Because transdiagnostic psychotherapies target shared mechanisms across EDs, evidence on their comparative performance may support more flexible and efficient treatment selection, particularly in settings where comorbidity and mixed symptom presentations are common. Beyond symptom improvement, the inclusion of treatment acceptability as a key outcome will further inform the feasibility of implementing these interventions in routine practice.

The results of this NMA may also inform future research priorities and guideline development. By mapping the current evidence network, identifying well-supported interventions, and highlighting uncertain or missing comparisons, this study may help direct future RCTs toward the most clinically relevant questions. Ultimately, we expect this work to contribute meaningful evidence for optimizing psychological treatment strategies for individuals with EDs.

## Appendix

Multimedia Appendix 1 Search strategies for retrieving studies on psychotherapy for anxiety-depression comorbidity.

## Abbreviations

ACT: acceptance and commitment therapy
BA: behavioral activation
BAI: Beck Anxiety Inventory
BDI-II: Beck Depression Inventory–II
CAU: care as usual
CBT: cognitive behavioral therapy
CINeMA: Confidence in Network Meta-Analysis
DS-CBT: disorder-specific cognitive behavioral therapy
EBP: evidence-based psychotherapy
ED: emotional disorder
ERT: emotion regulation therapy
GAD: generalized anxiety disorder
GAD-7: Generalized Anxiety Disorder–7
GRADE: Grading of Recommendations Assessment, Development, and Evaluation
HADS: Hospital Anxiety and Depression Scale
iCBT: internet-delivered cognitive behavioral therapy
LLM: large language model
MBCT: mindfulness-based cognitive therapy
NMA: network meta-analysis
PHQ-9: Patient Health Questionnaire–9
PRISMA: Preferred Reporting Items for Systematic Reviews and Meta-Analyses
PRISMA-NMA: Preferred Reporting Items for Systematic Reviews and Meta-Analyses extension for network meta-analyses
PST: problem-solving therapy
RCT: randomized controlled trial
RoB 2: version 2 of the Cochrane risk of bias tool for randomized trials
SAD: social anxiety disorder
SMD: standardized mean difference
TBT: transdiagnostic behavior therapy
TD-CBT: transdiagnostic cognitive behavioral therapy
UP: Unified Protocol

## Data Availability

The data and code will be made openly available (where licensing permits) via the Open Science Framework.
