# Supplementary material for: Comparative Efficacy and Acceptability of Transdiagnostic Psychotherapies for Emotional Disorders: Protocol for a Network Meta-Analysis of Randomized Controlled Trials
Source: JMIR Res Protoc. 2026 Oct 6;15:e104791. doi: 10.2196/104791 (PMC13684113; doi:10.2196/104791)
Supplement: Supplementary file 1 [file resprot_v15i1e104791_app1.docx]

**Multimedia Appendix 1. Search strategies for retrieving studies on psychotherapy for anxiety–depression comorbidity**

| **Database** | **Search strategy** |
| --- | --- |
| **PubMed** | (Psychotherapy[MH] OR psychotherap*[All Fields] OR cbt[All Fields] OR "behavior therapies"[All Fields] OR "behavior therapy"[All Fields] OR "behavior therapeutic"[All Fields] OR "behavior therapeutical"[All Fields] OR "behavior therapeutics"[All Fields] OR "behavior therapeutist"[All Fields] OR "behavior therapeutists"[All Fields] OR "behavior treatment"[All Fields] OR "behavior treatments"[All Fields] OR "behaviors therapies"[All Fields] OR "behaviors therapy"[All Fields] OR "behaviors therapeutics"[All Fields] OR "behaviors therapeutic"[All Fields] OR "behaviors therapeutical"[All Fields] OR "behaviors therapeutist"[All Fields] OR "behaviors therapeutists"[All Fields] OR "behaviors treatment"[All Fields] OR "behaviors treatments"[All Fields] OR "behavioral therapies"[All Fields] OR "behavioral therapy"[All Fields] OR "behavioral therapeutics"[All Fields] OR "behavioral therapeutic"[All Fields] OR "behavioral therapeutical"[All Fields] OR "behavioral therapeutist"[All Fields] OR "behavioral therapeutists"[All Fields] OR "behavioral treatment"[All Fields] OR "behavioral treatments"[All Fields] OR "behaviour therapies"[All Fields] OR "behaviour therapy"[All Fields] OR "behaviour therapeutic"[All Fields] OR "behaviour therapeutical"[All Fields] OR "behaviour therapeutics"[All Fields] OR "behaviour therapeutist"[All Fields] OR "behaviour therapeutists"[All Fields] OR "behaviour treatment"[All Fields] OR "behaviour treatments"[All Fields] OR "behaviours therapies"[All Fields] OR "behaviours therapy"[All Fields] OR "behaviours therapeutics"[All Fields] OR "behaviours therapeutic"[All Fields] OR "behaviours therapeutical"[All Fields] OR "behaviours therapeutist"[All Fields] OR "behaviours therapeutists"[All Fields] OR "behaviours treatment"[All Fields] OR "behaviours treatments"[All Fields] OR "behavioural therapies"[All Fields] OR "behavioural therapy"[All Fields] OR "behavioural therapeutics"[All Fields] OR "behavioural therapeutic"[All Fields] OR "behavioural therapeutical"[All Fields] OR "behavioural therapeutist"[All Fields] OR "behavioural therapeutists"[All Fields] OR "behavioural treatment"[All Fields] OR "behavioural treatments"[All Fields] OR "cognition therapies"[All Fields] OR "cognition therapie"[All Fields] OR "cognition therapy"[All Fields] OR "cognition therapeutical"[All Fields] OR "cognition therapeutic"[All Fields] OR "cognition therapeutics"[All Fields] OR "cognition therapeutist"[All Fields] OR "cognition therapeutists"[All Fields] OR "cognition treatment"[All Fields] OR "cognition treatments"[All Fields] OR psychodynamic[All Fields] OR Psychoanalysis[MH] OR psychoanalysis[All Fields] OR psychoanalytic*[All Fields] OR counselling[All Fields] OR counseling[All Fields] OR Counseling[MH] OR "problem-solving"[All Fields] OR mindfulness[All Fields] OR (acceptance[All Fields] AND commitment[All Fields] ) OR "assertiveness training"[All Fields] OR "behavior activation"[All Fields] OR "behaviors activation"[All Fields] OR "behavioral activation"[All Fields] OR "cognitive therapies"[All Fields] OR "cognitive therapy"[All Fields] OR "cognitive therapeutic"[All Fields] OR "cognitive therapeutics"[All Fields] OR "cognitive therapeutical"[All Fields] OR "cognitive therapeutist"[All Fields] OR "cognitive therapeutists"[All Fields] OR "cognitive treatment"[All Fields] OR "cognitive treatments"[All Fields] OR "cognitive restructuring"[All Fields] OR (("compassion-focused"[All Fields] OR "compassion-focussed"[All Fields]) AND (therapy[SH] OR therapies[All Fields] OR therapy[All Fields] OR therape*[All Fields] OR therapis*[All Fields] OR Therapeutics[All Fields] OR treatment*[All Fields])) OR ((therapy[SH] OR therapies[All Fields] OR therapy[All Fields] OR therape*[All Fields] OR therapis*[All Fields] OR Therapeutics[MH] OR treatment*[All Fields]) AND constructivist*[All Fields]) OR "metacognitive therapies"[All Fields] OR "metacognitive therapy"[All Fields] OR "metacognitive therapeutic"[All Fields] OR "metacognitive therapeutics"[All Fields] OR "metacognitive therapeutical"[All Fields] OR "metacognitive therapeutist"[All Fields] OR "metacognitive therapeutists"[All Fields] OR "metacognitive treatment"[All Fields] OR "metacognitive treatments"[All Fields] OR "meta-cognitive therapies"[All Fields] OR "meta-cognitive therapy"[All Fields] OR "meta-cognitive therapeutic"[All Fields] OR "meta-cognitive therapeutics"[All Fields] OR "meta-cognitive therapeutical"[All Fields] OR "meta-cognitive therapeutist"[All Fields] OR "meta-cognitive therapeutists"[All Fields] OR "meta-cognitive treatment"[All Fields] OR "meta-cognitive treatments"[All Fields] OR "solution-focused therapies"[All Fields] OR "solution-focused therapy"[All Fields] OR "solution-focused therapeutic"[All Fields] OR "solution-focused therapeutics"[All Fields] OR "solution-focused therapeutical"[All Fields] OR "solution focused therapies"[All Fields] OR "solution focused therapy"[All Fields] OR "solution focused therapeutic"[All Fields] OR "solution focused therapeutics"[All Fields] OR "solution focused therapeutical"[All Fields] OR "solution-focussed therapies"[All Fields] OR "solution-focussed therapy"[All Fields] OR "solution-focussed therapeutic"[All Fields] OR "solution-focussed therapeutics"[All Fields] OR "solution-focussed therapeutical"[All Fields] OR "solution focussed therapies"[All Fields] OR "solution focussed therapy"[All Fields] OR "solution focussed therapeutic"[All Fields] OR "solution focussed therapeutics"[All Fields] OR "solution focussed therapeutical"[All Fields] OR "self-control therapies"[All Fields] OR "self-control therapy"[All Fields] OR "self-control therapeutics"[All Fields] OR "self-control therapeutical"[All Fields] OR "self-control therapeutic"[All Fields] OR "self-control training"[All Fields] OR "self-control trainings"[All Fields] OR "self control therapies"[All Fields] OR "self control therapy"[All Fields] OR "self control therapeutics"[All Fields] OR "self control therapeutical"[All Fields] OR "self control therapeutic"[All Fields] OR "self control training"[All Fields] OR "self control trainings"[All Fields] OR exposure[All Fields] OR relaxation[All Fields] OR EMDR[All Fields] OR ("eye movement" and desensiti*[All Fields]) OR "panic management"[All Fields] OR "response prevention"[All Fields] OR ERP[All Fields] OR transdiagnostic[All Fields] OR trans-diagnostic[All Fields] OR cross-diagnostic[All Fields] OR "Unified Protocol"[All Fields] OR UP[All Fields] OR "TD-iCBT"[All Fields] OR "transdiagnostic internet-based CBT"[All Fields] OR "TD-CBT"[All Fields] OR "TD CBT"[All Fields] OR "Emotion Regulation Therapy"[All Fields] OR ERT[All Fields] OR "Transdiagnostic Behavior Therapy"[All Fields] OR TBT[All Fields] OR "Affect Regulation Training"[All Fields] OR ART[All Fields] OR "False Safety Behavior Elimination Therapy"[All Fields] OR "F-SET"[All Fields] OR "Emotion Regulation Training"[All Fields] OR ERT[All Fields] OR "Coordinated Anxiety Learning and Management"[All Fields] OR CALM[All Fields])  AND  ("social anxiety"[All Fields] OR shy[All Fields] OR ("shyness"[MeSH Terms] OR "shyness"[All Fields]) OR "test anxiety"[All Fields] OR gad[All Fields] OR "generalized anxiety"[All Fields] OR "generalised anxiety"[All Fields] OR worry[All Fields] OR ("panic"[MeSH Terms] OR "panic"[All Fields]) OR (agoraphobi[All Fields] OR agoraphobia[All Fields] OR agoraphobia'[All Fields] OR agoraphobia's[All Fields] OR agoraphobias[All Fields] OR agoraphobic[All Fields] OR agoraphobic's[All Fields] OR agoraphobics[All Fields] OR agoraphobics'[All Fields] OR agoraphobie[All Fields] OR agoraphobien[All Fields]) OR "anxiety disorder"[All Fields] OR "social phobia"[All Fields] OR "social anxiety disorder"[All Fields] )  AND  ("randomized controlled trial" OR "randomised controlled trial" OR "controlled clinical trial" OR "randomized" OR "randomised" OR "placebo" OR "clinical trial*" OR "trial*" OR "RCT") |
| **Embase** | 'psychotherapy'/exp OR psychotherap* OR 'psychotherapy' OR 'psychotherapy' OR 'psychotherapies' OR 'psychotherapeutic' OR 'psychotherapeutical' OR 'psychotherapeutics' OR 'Behavior Therapy'/exp OR 'Cognitive Behavioral Therapy'/exp OR 'CBT' OR 'behavior therapies' OR 'behavior therapy' OR 'behavior therapeutic' OR 'behavior therapeutical' OR 'behavior therapeutics' OR 'behavior therapeutist' OR 'behavior therapeutists' OR 'behavior treatment' OR 'behavior treatments' OR 'behaviors therapies' OR 'behaviors therapy' OR 'behaviors therapeutics' OR 'behaviors therapeutic' OR 'behaviors therapeutical' OR 'behaviors therapeutist' OR 'behaviors therapeutists' OR 'behaviors treatment' OR 'behaviors treatments' OR 'behavioral therapies' OR 'behavioral therapy' OR 'behavioral therapeutics' OR 'behavioral therapeutic' OR 'behavioral therapeutical' OR 'behavioral therapeutist' OR 'behavioral therapeutists' OR 'behavioral treatment' OR 'behavioral treatments' OR 'behaviour therapies' OR 'behaviour therapy' OR 'behaviour therapeutic' OR 'behaviour therapeutical' OR 'behaviour therapeutics' OR 'behaviour therapeutist' OR 'behaviour therapeutists' OR 'behaviour treatment' OR 'behaviour treatments' OR 'behaviours therapies' OR 'behaviours therapy' OR 'behaviours therapeutics' OR 'behaviours therapeutic' OR 'behaviours therapeutical' OR 'behaviours therapeutist' OR 'behaviours therapeutists' OR 'behaviours treatment' OR 'behaviours treatments' OR 'behavioural therapies' OR 'behavioural therapy' OR 'behavioural therapeutics' OR 'behavioural therapeutic' OR 'behavioural therapeutical' OR 'behavioural therapeutist' OR 'behavioural therapeutists' OR 'behavioural treatment' OR 'behavioural treatments' OR 'cognition therapies' OR 'cognition therapie' OR 'cognition therapy' OR 'cognition therapeutical' OR 'cognition therapeutic' OR 'cognition therapeutics' OR 'cognition therapeutist' OR 'cognition therapeutists' OR 'cognition treatment' OR 'cognition treatments' OR 'cognitive therapies' OR 'cognitive therapy' OR 'cognitive therapeutic' OR 'cognitive therapeutics' OR 'cognitive therapeutical' OR 'cognitive therapeutist' OR 'cognitive therapeutists' OR 'cognitive treatment' OR 'cognitive treatments' OR 'cognitive restructuring' OR 'Emotion Focused Therapy'/exp OR 'Psychoanalysis'/exp OR 'psychoanalysis' OR 'psychoanalytic' OR 'psychoanalytical' OR 'Psychodynamic Psychotherapy'/exp OR 'psychodynamic' OR 'Psychotherapeutic Counseling'/exp OR 'counselling' OR 'counseling' OR 'problem-solving' OR 'problem solving' OR 'mindfulness' OR 'acceptance and commitment' OR 'assertiveness training' OR 'behavior activation' OR 'behaviors activation' OR 'behavioral activation' OR 'behaviour activation' OR 'behaviours activation' OR 'behavioural activation' OR 'metacognitive therapies' OR 'metacognitive therapy' OR 'metacognitive therapeutic' OR 'metacognitive therapeutics' OR 'metacognitive therapeutical' OR 'metacognitive therapeutist' OR 'metacognitive therapeutists' OR 'metacognitive treatment' OR 'metacognitive treatments' OR 'meta-cognitive therapies' OR 'meta-cognitive therapy' OR 'meta-cognitive therapeutic' OR 'meta-cognitive therapeutics' OR 'meta-cognitive therapeutical' OR 'meta-cognitive therapeutist' OR 'meta-cognitive therapeutists' OR 'meta-cognitive treatment' OR 'meta-cognitive treatments' OR 'Solution Focused Therapy'/exp OR 'solution-focused therapies' OR 'solution-focused therapy' OR 'solution-focused therapeutic' OR 'solution-focused therapeutics' OR 'solution-focused therapeutical' OR 'solution-focussed therapies' OR 'solution-focussed therapy' OR 'solution-focussed therapeutic' OR 'solution-focussed therapeutics' OR 'solution-focussed therapeutical' OR 'solution focused therapies' OR 'solution focused therapy' OR 'solution focused therapeutic' OR 'solution focused therapeutics' OR 'solution focused therapeutical' OR 'solution focussed therapies' OR 'solution focussed therapy' OR 'solution focussed therapeutic' OR 'solution focussed therapeutics' OR 'solution focussed therapeutical' OR 'self-control therapies' OR 'self-control therapy' OR 'self-control therapeutics' OR 'self-control therapeutical' OR 'self-control therapeutic' OR 'self-control training' OR 'self-control trainings' OR 'self control therapies' OR 'self control therapy' OR 'self control therapeutics' OR 'self control therapeutical' OR 'self control therapeutic' OR 'self control training' OR 'self control trainings' OR 'compassion-focused' OR 'compassion-focussed' OR 'compassion focused' OR 'compassion focussed' OR 'exposure' OR 'relaxation' OR 'EMDR' OR 'eye movement and reprocessing' OR 'panic management' OR 'response prevention' OR 'ERP' OR 'transdiagnostic' OR 'trans-diagnostic' OR 'cross-diagnostic' OR 'Unified Protocol' OR 'transdiagnostic internet-based CBT' OR 'Emotion Regulation Therapy' OR 'Transdiagnostic Behavior Therapy' OR 'Affect Regulation Training' OR 'False Safety Behavior Elimination Therapy' OR 'Emotion Regulation Training' OR 'Coordinated Anxiety Learning and Management'  AND  'anxiety disorder'/exp OR 'anxiety disorder' OR 'generalized anxiety disorder'/exp OR 'generalized anxiety disorder' OR 'generalised anxiety disorder'/exp OR 'gad' OR 'generalised anxiety disorder' OR 'generalized anxiety' OR 'generalised anxiety' OR 'worry' OR 'social phobia'/exp OR 'social phobia' OR 'social anxiety disorder'/exp OR 'social anxiety disorder' OR 'social anxiety' OR 'acute stress disorder'/exp OR 'acute stress disorder' OR 'acute stress' OR 'panic'/exp OR 'panic' OR 'agorophobia'  AND  'randomized controlled trial'/exp OR 'controlled clinical trial'/exp OR 'clinical trial'/exp OR 'randomized' OR 'randomised' OR 'placebo' OR 'RCT' OR 'random allocation' OR 'randomly assigned' |
| **PsycINFO** | (DE "Psychotherapy" OR "Psychotherapy" OR "psychotherapies" OR "psychotherapeutic" OR "psychotherapeutical" OR "psychotherapeutics" OR DE "Behavior Therapy" OR DE "Cognitive Behavior Therapy" OR "CBT" OR "behavior therapies" OR "behavior therapy" OR "behavior therapeutic" OR "behavior therapeutical" OR "behavior therapeutics" OR "behavior therapeutist" OR "behavior therapeutists" OR "behavior treatment" OR "behavior treatments" OR "behaviors therapies" OR "behaviors therapy" OR "behaviors therapeutics" OR "behaviors therapeutic" OR "behaviors therapeutical" OR "behaviors therapeutist" OR "behaviors therapeutists" OR "behaviors treatment" OR "behaviors treatments" OR "behavioral therapies" OR "behavioral therapy" OR "behavioral therapeutics" OR "behavioral therapeutic" OR "behavioral therapeutical" OR "behavioral therapeutist" OR "behavioral therapeutists" OR "behavioral treatment" OR "behavioral treatments" OR "behaviour therapies" OR "behaviour therapy" OR "behaviour therapeutic" OR "behaviour therapeutical" OR "behaviour therapeutics" OR "behaviour therapeutist" OR "behaviour therapeutists" OR "behaviour treatment" OR "behaviour treatments" OR "behaviours therapies" OR "behaviours therapy" OR "behaviours therapeutics" OR "behaviours therapeutic" OR "behaviours therapeutical" OR "behaviours therapeutist" OR "behaviours therapeutists" OR "behaviours treatment" OR "behaviours treatments" OR "behavioural therapies" OR "behavioural therapy" OR "behavioural therapeutics" OR "behavioural therapeutic" OR "behavioural therapeutical" OR "behavioural therapeutist" OR "behavioural therapeutists" OR "behavioural treatment" OR "behavioural treatments" OR "cognition therapies" OR "cognition therapie" OR "cognition therapy" OR "cognition therapeutical" OR "cognition therapeutic" OR "cognition therapeutics" OR "cognition therapeutist" OR "cognition therapeutists" OR "cognition treatment" OR "cognition treatments" OR "cognitive therapies" OR "cognitive therapy" OR "cognitive therapeutic" OR "cognitive therapeutics" OR "cognitive therapeutical" OR "cognitive therapeutist" OR "cognitive therapeutists" OR "cognitive treatment" OR "cognitive treatments" OR "cognitive restructuring" OR DE "Emotion Focused Therapy" OR DE "Psychoanalysis" OR "psychoanalysis" OR "psychoanalytic" OR "psychoanalytical "OR DE "Psychodynamic Psychotherapy" OR "psychodynamic" OR DE "Psychotherapeutic Counseling" OR "counselling" OR "counseling" OR "problem-solving" OR "problem solving" OR "mindfulness" OR "acceptance and commitment" OR "assertiveness training" OR "behavior activation" OR "behaviors activation" OR "behavioral activation" OR "behaviour activation" OR "behaviours activation" OR "behavioural activation" OR "metacognitive therapies" OR "metacognitive therapy" OR "metacognitive therapeutic" OR "metacognitive therapeutics" OR "metacognitive therapeutical" OR "metacognitive therapeutist" OR "metacognitive therapeutists" OR "metacognitive treatment" OR "metacognitive treatments" OR "meta-cognitive therapies" OR "meta-cognitive therapy" OR "meta-cognitive therapeutic" OR "meta-cognitive therapeutics" OR "meta-cognitive therapeutical" OR "meta-cognitive therapeutist" OR "meta-cognitive therapeutists" OR "meta-cognitive treatment" OR "meta-cognitive treatments" OR DE "Solution Focused Therapy" OR "solution-focused therapies" OR "solution-focused therapy" OR "solution-focused therapeutic" OR "solution-focused therapeutics" OR "solution-focused therapeutical" OR "solution-focussed therapies" OR "solution-focussed therapy" OR "solution-focussed therapeutic" OR "solution-focussed therapeutics" OR "solution-focussed therapeutical" OR "solution focused therapies" OR "solution focused therapy" OR "solution focused therapeutic" OR "solution focused therapeutics" OR "solution focused therapeutical" OR "solution focussed therapies" OR "solution focussed therapy" OR "solution focussed therapeutic" OR "solution focussed therapeutics" OR "solution focussed therapeutical" OR "self-control therapies" OR "self-control therapy" OR "self-control therapeutics" OR "self-control therapeutical" OR "self-control therapeutic" OR "self-control training" OR "self-control trainings" OR "self control therapies" OR "self control therapy" OR "self control therapeutics" OR "self control therapeutical" OR "self control therapeutic" OR "self control training" OR "self control trainings" OR "compassion-focused" OR "compassion-focussed" OR "compassion focused" OR "compassion focussed" OR "therapies" OR "therapy" OR "therapie" OR "therapist" OR "therapists" OR "therapeut" OR "treatment" OR "treatments" OR "constructivist" OR "therapies" OR "therapy" OR "therapie" OR "therapist" OR "therapists" OR "therapeut" OR "treatment" OR "treatments" OR "exposure" OR "relaxation" OR "EMDR" OR "eye movement and reprocessing" OR "panic management" OR "response prevention" OR "ERP" OR "transdiagnostic" OR "trans-diagnostic" OR "cross-diagnostic" OR "Unified Protocol" OR "UP" OR "TD-iCBT" OR "transdiagnostic internet-based CBT" OR "TD-CBT" OR "TD CBT" OR "Emotion Regulation Therapy" OR "ERT" OR "Transdiagnostic Behavior Therapy" OR "TBT" OR "Affect Regulation Training" OR "ART" OR "False Safety Behavior Elimination Therapy" OR "F-SET" OR "Emotion Regulation Training" OR "ERT" OR "Coordinated Anxiety Learning and Management" OR "CALM"  AND  "social anxiety" OR "shy" OR "shyness" OR "test anxiety" OR "gad" OR "generalized anxiety" OR "generalised anxiety" OR "worry" OR "panic" OR "agoraphobi*" OR "anxiety disorder" OR "anxiety disorders" OR "SAD" OR "social phobia" OR "social anxiety disorder"  )  AND  ("randomized controlled trial" OR "randomised controlled trial" OR "controlled clinical trial" OR "randomized" OR "randomised" OR "placebo" OR "clinical trial*" OR "trial*" OR "RCT") |
| **Cochrane Library** | IDSearch  #1"social anxiety disorder":ti,ab,kw  #2"GAD":ti,ab,kw  #3"social anxiety":ti,ab,kw  #4"test anxiety":ti,ab,kw  #5"generalised anxiety disorder":ti,ab,kw  #6"generalised anxiety":ti,ab,kw  #7"worry":ti,ab,kw  #8"panic":ti,ab,kw  #9"panic disorder":ti,ab,kw  #10"agoraphobia":ti,ab,kw  #11"phobia":ti,ab,kw  #12#1 OR #2 OR #3 OR #4 OR #5 OR #6 OR #7 OR #8 OR #9 OR #10 OR #11  #13"psychotherapy":kw  #14"CBT":ti,ab,kw  #15cognitive behavi* therap*:ti,ab,kw  #16psychodynamic:ti,ab,kw  #17"psychoanalysis":ti,ab,kw  #18psychoanalys*:ti,ab,kw  #19"counseling":ti,ab,kw  #20"problem solving":ti,ab,kw  #21"acceptance and commitment":ti,ab,kw  #22"assertiveness training":ti,ab,kw  #23"behavioral activation":ti,ab,kw  #24"behavioural activation":ti,ab,kw  #25"mindfulness":ti,ab,kw  #26"metacognitive therap*":ti,ab,kw  #27"solution focused therap*":ti,ab,kw  #28"self-control therap*":ti,ab,kw  #29"self-control training":ti,ab,kw  #30"exposure":ti,ab,kw  #31"relaxation":ti,ab,kw  #32"EMDR":ti,ab,kw  #33"eye movement desensitization":ti,ab,kw  #34"panic management":ti,ab,kw  #35"response prevention":ti,ab,kw  #36 "transdiagnostic":ti,ab,kw  #37 "unified protocol":ti,ab,kw  #38 "transdiagnostic behavior therap*":ti,ab,kw  #39 "emotion regulation therap*":ti,ab,kw  #40 "transdiagnostic internet":ti,ab,kw  #41 "TD-iCBT":ti,ab,kw  #42 "unified transdiagnostic":ti,ab,kw  #43 "affect regulation training":ti,ab,kw  #44 "emotional disorder*":ti,ab,kw  #45 #13 OR #14 OR #15 OR #16 OR #17 OR #18 OR #19 OR #20 OR #21 OR #22 OR #23 OR #24 OR #25 OR #26 OR #27 OR #28 OR #29 OR #30 OR #31 OR #32 OR #33 OR #34 OR #35 OR #37 OR #38 OR #39 OR #40 OR #41 OR #42 OR #43 OR #44  #46 #12 AND #45 in Trials |
